# Presence of Myeloid Mutations in Patients with Chronic Myeloid Leukemia Increases Risk of Cardiovascular Event on Tyrosine Kinase Inhibitor Treatment

**DOI:** 10.3390/cancers15133384

**Published:** 2023-06-28

**Authors:** Ruth Stuckey, Adrián Segura-Díaz, María Nieves Sáez Perdomo, Manuel Mateo Pérez Encinas, Jóse David González San Miguel, Yanira Florido, Santiago Sánchez-Sosa, Juan Francisco López-Rodríguez, Cristina Bilbao-Sieyro, María Teresa Gómez-Casares

**Affiliations:** 1 Hematology Department, Hospital Universitario de Gran Canaria Dr. Negrín, 35019 Las Palmas de Gran Canaria, Spain; rstuckey@fciisc.es (R.S.); asegdia@gobiernodecanarias.org (A.S.-D.); floryyana@hotmail.com (Y.F.); jsanchez@fciisc.es (S.S.-S.); juanfra5226@gmail.com (J.F.L.-R.); 2 Diagnóstica Longwood, 50011 Zaragoza, Spain; mnieves.saez@dlongwood.com; 3 Hematology Department, Hospital Clínico Universitario de Santiago de Compostela, 15706 Santiago de Compostela, Spain; m.p.encinas@gmail.com; 4 Hematology Department, Hospital Universitario Insular de Gran Canaria, 35016 Las Palmas de Gran Canarias, Spain; jdgonsan@gobiernodecanarias.org; 5 Morphology Department, Universidad de Las Palmas de Gran Canaria, 35016 Las Palmas de Gran Canaria, Spain; 6 Department of Medical Sciences, Universidad de Las Palmas de Gran Canaria, 35016 Las Palmas de Gran Canaria, Spain

**Keywords:** chronic myeloid leukemia (CML), cardiovascular event, prognosis, somatic mutation, tyrosine kinase inhibitor (TKI), next-generation sequencing (NGS), clonal hematopoiesis of indeterminate potential (CHIP)

## Abstract

**Simple Summary:**

Tyrosine kinase inhibitors (TKI), such as imatinib, nilotinib, and dasatinib, are the first-line treatment of choice for patients with chronic myeloid leukemia (CML). However, patients may develop serious cardiovascular events (CVE) with their use. Cardiotoxicity is an important issue given the associated mortality of CVE and the long-term nature of TKI treatment. This study aimed to investigate the association of the presence of somatic myeloid mutations (including those with a reported role in clonal hematopoiesis) at diagnosis and the development of CVE for 102 patients on TKI treatment. The presence of a somatic myeloid mutation was a significant risk factor for CVE on any TKI and shortened the CV event-free survival for patients receiving first-line imatinib treatment. Our work shows that the low risk of CVE, traditionally associated with first-line imatinib, was increased by the presence of myeloid mutations as well as older age. Our findings may help inform first-line TKI choice.

**Abstract:**

For chronic myeloid leukemia (CML) patients with a known risk of cardiovascular events (CVE), imatinib is often recommended for first-line tyrosine kinase inhibitor (TKI) treatment rather than a second-generation TKI (2G-TKI) such as nilotinib or dasatinib. To date, very few studies have evaluated the genetic predisposition associated with CVE development on TKI treatment. In this retrospective study of 102 CML patients, 26 CVEs were reported during an average follow-up of over 10 years. Next-generation sequencing identified pathogenic/likely pathogenic mutations in genes associated with myeloid malignancies in 24.5% of the diagnostic samples analyzed. Patients with a recorded CVE had more myeloid mutations (0.48 vs. 0.14, *p* = 0.019) and were older (65.1 vs. 55.7 years, *p* = 0.016). Age ≥ 60 years and receiving a 2G-TKI in first-line were CVE risk factors. The presence of a pathogenic somatic myeloid mutation was an independent risk factor for CVE on any TKI (HR 2.79, *p* = 0.01), and significantly shortened the CV event-free survival of patients who received first-line imatinib (by 70 months, *p* = 0.011). Indeed, 62% of patients on imatinib with mutations had a CVE vs. the 19% on imatinib with a mutation and no CVE. In conclusion, myeloid mutations detectable at diagnosis increase CVE risk, particularly for patients on imatinib, and might be considered for first-line TKI choice.

## 1. Introduction

Chronic myeloid leukemia (CML) is a hematopoietic disorder characterized by the excessive proliferation and uncontrolled clonal expansion of myeloid cells and accounts for approximately 15% of leukemias diagnosed in adults [1]. The hallmark of CML is the presence of a *BCR::ABL1* gene fusion due to the balanced chromosomal translocation (9;22) (q34;q11) [2,3]. This genetic alteration leads to the production of an oncoprotein BCR::ABL1 with constitutively active ABL1 tyrosine kinase activity, whose deregulated action is responsible for the development of this myeloproliferative neoplasm.

Tyrosine kinase inhibitors (TKI) are the first-line treatment of choice for patients with CML [4]. Imatinib—now known as a first-generation TKI—was designed to specifically inhibit the BCR::ABL1 tyrosine kinase. To date, four TKIs have been approved for the first-line treatment of chronic phase CML (CP-CML): imatinib, and the second-generation TKIs (2G-TKI) dasatinib, nilotinib, and bosutinib. The third-generation TKI ponatinib is also approved for CML patients who are resistant to nilotinib or dasatinib and for patients with the T315I mutation.

Randomized trials have demonstrated that the second- and third-generation TKIs have potent antileukemic effects in patients resistant to imatinib, as well as a superior efficacy compared to imatinib in the first line [5,6,7,8]. One disadvantage associated with their use is the development of serious cardiovascular side effects such as arterial occlusion (AOE) and venous thromboembolism (VTE) events. Clinically relevant TKI treatment-related cardiovascular events (CVE) include coronary atherothrombotic, cerebrovascular, and peripheral arterial events associated with nilotinib [9]; arterial and venous occlusive events with ponatinib [10,11]; and pulmonary hypertension with dasatinib use [12,13].

The exact incidence of these events is still subject to debate, but most studies are in accordance that the incidence of AOE and VTE increases with the length of treatment; with a higher TKI dose; and with pre-existing cardiovascular risk factors, such as a history of prior CV events, advanced age, and comorbidities including arterial hypertension, hyperlipidemia, diabetes mellitus, etc. [14].

The development of new technologies, such as next-generation sequencing (NGS), has led to major advances in the understanding of the molecular pathogenesis of myeloid neoplasias [15]. For example, an accumulation of somatic mutations has been shown to confer a selective advantage and lead to clonal hematopoiesis of indeterminate potential (CHIP) in otherwise healthy individuals, a phenomenon associated with advanced age [16]. The most frequently mutated genes in CHIP are the DTA genes (*DNMT3A, TET2, ASXL1*) and *JAK2*. CHIP confers a higher propensity of developing a myeloid neoplasm as well as an association with cardiovascular diseases and shorter overall survival [16,17,18].

Until a few years ago, CP-CML was considered to be a genetically homogeneous disease. However, in 2017, Kim et al. showed that CHIP-related mutations can be present at diagnosis [19]. This finding of mutated cancer-related genes at diagnosis has been confirmed by other groups and, importantly, has been shown to be associated with poor outcomes, including an inferior response to TKI, progression to blast crisis, and reduced treatment-free remission (TFR) after TKI discontinuation [20,21,22,23,24].

To date, very few studies have evaluated the genetic predisposition associated with the development of AOE/VTE induced by TKIs in CML. The aim of this work was to determine the mutational profile of a series of CML patients and investigate the association between the presence of somatic myeloid mutations (including those with a reported role in CHIP) and the development of CVE on TKI treatment. This is an important objective in the CML field since TKI exposure is long and patients are often young at diagnosis and the start of TKI treatment.

## 2. Materials and Methods

### 2.1. Patients

Patients aged 18 years and above with a confirmed CML diagnosis, who had received treatment with TKI with a minimum follow-up of 3 years, were consecutively recruited at three participating hospitals. All medical care was provided at the CML-managing hospital, including follow-up with cardiologists, according to the routine standard of practice. For the included patients, data were retrospectively collected on the TKI treatment received and thromboembolic or chronic ischemic events identified from the patient’s medical history. The following CVEs during TKI treatment were considered: myocardial infarction, acute coronary syndrome, peripheral artery disease, chronic ischemic syndrome, ischemic stroke, transient ischemic attack, carotid atherosclerosis, deep vein thrombosis, pulmonary embolism, and portal vein thrombosis. No patients were lost to follow-up during the observation period.

For the case-control study (*n* = 42), two homogenous groups of 21 age- and gender-matched CML patients were formed from the discovery cohort with (case) and without CVE (control) in their medical history.

### 2.2. Next-Generation Sequencing (NGS)

We retrospectively analyzed 200 ng of genomic DNA extracted from peripheral blood at diagnosis. Diagnostic samples were selected for sequencing to enable the identification of mutations in CML leukemic cells since TKI treatment can cause the gain or loss of somatic mutations [19,25] and samples taken when patients are in molecular remission typically have undetectable levels of leukemia cells [19,24].

NGS was performed with the MiSeq (Illumina, San Diego, CA, USA) platform using the targeted 30-gene panel Myeloid Solution™ (SOPHiA GENETICS, Saint-Sulpice, Switzerland) with an average coverage (read depth) > 3500 reads. Variants were identified using the analysis software SOPHiA DDM (4.8.1.3). Only variants with an allelic frequency (VAF) ≥ 2%, a described population frequency (MAF) < 1%, and an annotated pathogenic effect (or likely pathogenic) were included [26], with pathogenicity determined according to the Association for Molecular Pathology guidelines [27]. Variants with a VAF of 40–60% were excluded from statistical analysis due to potential germline origin.

### 2.3. Statistical Analysis

Data normality was determined using the Kolmogorov–Smirnov test. Incidence density was calculated in events/person-year by dividing the total number of CVEs by the total time of follow-up from diagnosis of all patients. Chi-squared univariable tests and the Cox regression multivariable analyses analyzed the impact of the independent variables on the risk of CVEs. Differences between the two groups were determined using a student-paired *t*-test of equal variance for the unpaired samples. CV event-free survival rate was measured from the time of CML diagnosis until the event or date of the last follow-up. Survival probabilities were estimated using the Kaplan–Meier method and the log-rank test was used for statistical comparison. *p*-values <0.05 were considered statistically significant. Analyses were performed using the SPSS statistical software, version 22.0.

## 3. Results

### 3.1. Patient Characteristics

The cohort included 102 CML patients, 61 of which were male (59.8%), with an average age at diagnosis of 58.1 years (the patient characteristics are shown in Supplementary Table S1). With a median post-diagnosis follow-up of 131 months (range 36.4–228.9 months), two patients progressed to the advanced or blast phase (2.0%), while one was diagnosed in the blast phase.

The first-line TKI treatment received was imatinib for 72 patients (70.6%), nilotinib for 23 (22.5%), and dasatinib for seven patients (6.9%). The median TKI exposure (including all TKI switches) was 7.3 years (range 3.0–22.3 years). Fifteen patients (*n* = 15, 14.7%) received three or more different TKI. Bosutinib and ponatinib were received by only one patient each in the second line (and none in the first line).

### 3.2. Cardiovascular Events

A total of 39 patients (38.2%) had a CVE in their medical history, of these, 18 (17.6%) were events prior to the start of TKI and 26 were events on TKI treatment (25.5% of the CML cohort), 21 corresponding to AOE (80.8%) and 5 to VTE (19.2%, Table 1). The most frequent AOE induced by TKI were acute coronary syndrome (*n* = 11) and ischemic stroke (*n* = 5).

Of the patients who experienced a CVE, the median time to an AOE/VTE event was 4.3 years on TKI (range 9–191 months), with an incidence density of 0.159 CVE/person-years. Three patients (11.1%) had a second CVE while on TKI treatment. All patients who experienced a CVE on TKI had at least one traditional CV risk factor (high body mass index, active smoker, high arterial hypertension, or diabetes mellitus).

CVEs were registered for 18.1% of patients who received first-line imatinib (13/72), 39.1% of patients who received first-line nilotinib (9/23), and 42.9% of patients who received dasatinib as first-line TKI (3/7). Of the 26 events registered, 19 were experienced by patients receiving a first-line, four a second-line, and two a third-line TKI (both nilotinib). At the time of the event, eight patients were receiving imatinib (32%), 12 nilotinib (48%), and five dasatinib (20%); one patient had discontinued TKI treatment before the CVE (Supplementary Table S2). In the univariable analysis, imatinib at the time of the event was protective (OR 0.33, *p* = 0.017), while 2G-TKI at the time of the event was a risk factor for CVE (OR 2.44, *p* = 0.048; Supplementary Table S3).

When considering the time on first-line TKI to CVE, the median CV event-free survival rate for imatinib was not reached, for nilotinib was 120.5 months, and for dasatinib, the median was not reached (*p* = 0.018, Supplementary Figure S1). Patients who received first-line dasatinib were predominantly male and 28.6% had a prior CVE in their medical history (Supplementary Table S4).

### 3.3. Mutations

**Figure 1:**
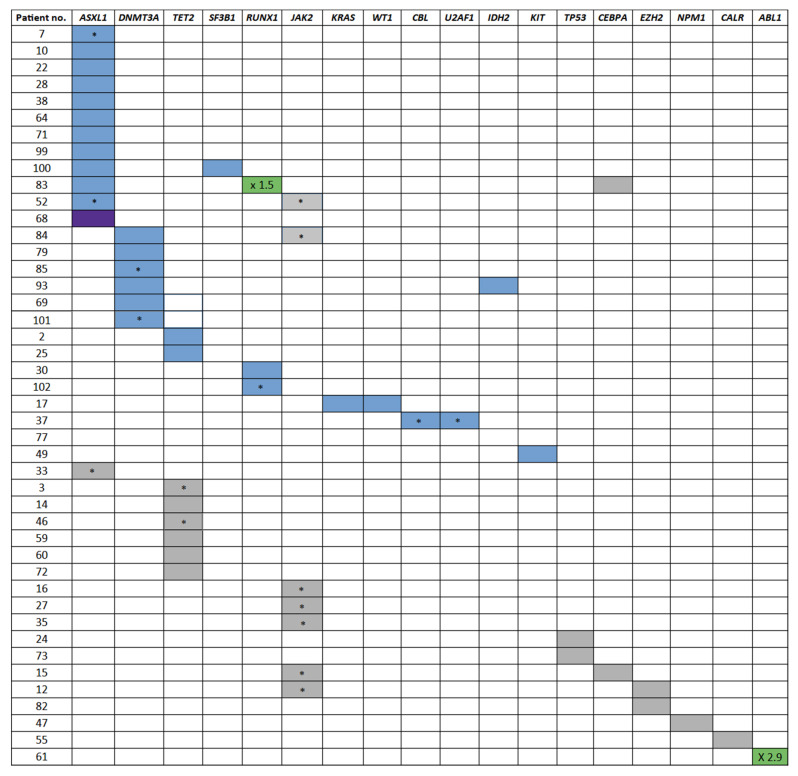
Mutations detected in the series of 102 CML patients. Variants detected in genes covered by the targeted myeloid panel (listed along the top) are represented by a blue square (pathogenic and likely pathogenic), gray square (additional mutations, including variants of uncertain significance), green square (copy number variations), or white square (no variant was detected). Darker shading signifies that two variants were detected in the same gene. An asterisk represents a potential germline variant.

In 77 patients (75.5%), no mutation was detected while 25 patients (24.5%) presented at least one pathogenic mutation (defined as pathogenic or likely pathogenic; Figure 1, blue squares). Forty-one patients presented at least one mutation (40.2%) when variants of uncertain significance (VUS) were also considered (Figure 1, gray squares).

Mutations were detected in 18 different genes from the myeloid panel, with an average allelic frequency (VAF) of 26.3%. The most frequently mutated genes were genes associated with CHIP: *ASXL1* (*n* = 12), *DNMT3A* (*n* = 5), and *TET2* (*n* = 3), while *JAK2* mutations were identified in seven patients (two pathogenic and five VUS; all detected variants are listed in Supplementary Table S5). Fifteen patients had a mutation with a VAF of 40–60%, suggesting they might be germline.

There was no difference in the age at diagnosis of patients harboring a pathogenic mutation vs. no pathogenic mutation (57.6 years vs. 58.3 years, *p* = 0.856) or any mutation (including VUS, 60.2 years vs. 55.0 years, *p* = 0.137 Student *t*-test). Of the 12 patients with an *ASXL1* mutation, seven (58.3%) were aged under 60 years.

### 3.4. Risk Factors for Cardiovascular Events

Patients who had a CVE on TKI were significantly older than patients who did not develop a CVE (65.1 vs. 55.7 years, respectively, *p* = 0.016) and the CV event-free survival rate was significantly shorter for patients aged ≥ 60 years (results not shown). Age was confirmed as a risk factor for CVE on TKI by the univariable and multivariable analysis (Table 2), while a previous CVE in the medical history was not a risk factor (Supplementary Table S6).

Mutations in *BCR::ABL1* were evaluated in only 13 patients (*BCR::ABL1* mutations were not studied in the majority of patients because the major molecular response was maintained), with three patients positive for a mutation and 10 negative. One of the three patients with a *BCR::ABL1* mutation had a myocardial infarction prior to the start of TKI. No association was detected between the presence of *BCR::ABL1* mutation and CVE on TKI.

Patients with a CVE in their medical history (*n* = 39) presented a higher incidence of pathogenic myeloid mutations (48.0% vs. 35.1%); however, the associations between the presence of any pathogenic mutation, CHIP mutation, or any mutation and the development of a CVE were not statistically significant when events prior to the start of TKI were included (*p* = 0.344, *p* = 0.435 and *p* = 0.407, respectively; Supplementary Table S7). In contrast, the association between a CVE and the presence of a pathogenic mutation (OR 3.25, *p* = 0.019), or any mutation (OR 2.62, *p* = 0.040), were significant and marginal for the CHIP mutation (OR 2.63, *p* = 0.082, Supplementary Table S6) for a CVE while on TKI treatment. No associations were found between a CVE and mutation in individual genes (results not shown). The presence of a pathogenic somatic myeloid mutation was not a risk factor for a CVE for patients receiving imatinib at the time of the CVE (OR 1.9, *p* = 0.409) but was of borderline significance for patients receiving a 2G-TKI at the time of the CVE (OR 3.11, *p* = 0.072; Supplementary Table S3).

A cause-specific hazard approach was used to confirm the risk factors identified from the univariable analyses for the development of a CVE while on TKI treatment. Neither imatinib nor 2G-TKI, at the time of a CVE, reached statistical significance (results not shown). On the other hand, the Cox regression multivariable analysis corroborated age ≥ 60 years and first-line nilotinib, or 2G-TKI, as CVE risk factors while imatinib was protective (OR 0.29, *p* = 0.002, Table 2).

Moreover, pathogenic somatic mutation or any somatic myeloid mutation were confirmed as independent risk factors (Table 2). Specifically, of the 72 patients who received first-line imatinib, 61.5% had at least one pathogenic somatic myeloid mutation and a CVE compared to just 18.6% of patients with a mutation and no CVE (Supplementary Table S8). Thus, the protective effect of imatinib in the first line was lost in patients with a pathogenic somatic mutation, i.e., a pathogenic somatic myeloid mutation was a significant risk factor for a CVE induced during first-line imatinib treatment (OR 2.93, CI: 1.14–7.57, *p* = 0.003).

To confirm these results, two homogeneous groups of 21 gender- and age-matched CML patients were formed with (case) and without CVE (control) in their medical history to exclude selection bias. The group of patients with CVE had a higher number of additional or pathogenic somatic myeloid mutations (0.62 vs. 0.24, *p* = 0.012; and 0.48 vs. 0.14, *p* = 0.019, respectively) and were more likely to have received nilotinib in the first line (*p* = 0.030) or a 2G-TKI in first or second line (*p* = 0.028, Supplementary Table S9). The Cox multivariable analyses confirmed the presence at diagnosis of any somatic myeloid mutation as a significant risk factor for patients receiving first-line imatinib or first-line 2G-TKI (*p* = 0.004, Supplementary Table S10), independently of age.

### 3.5. CV Event-Free Survival Rate

**Figure 2:**
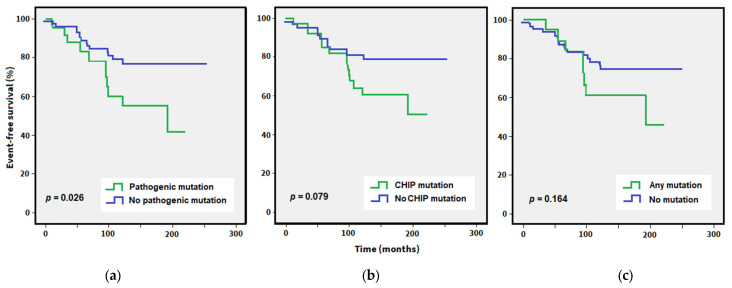
Kaplan–Meier curves of cardiovascular event-free survival for the whole cohort comparing patients with (green line) or without (blue line) a somatic myeloid mutation. Data were stratified according to the presence of (**a**) pathogenic mutation, (**b**) CHIP mutation, or (**c**) any additional somatic mutation (including variants of uncertain significance). Time is calculated from the date of diagnosis to the date of a cardiovascular event or the date of the last follow-up. Significance is determined using the log-rank test.

The negative effect of a pathogenic somatic myeloid mutation on a CVE while on TKI was also apparent in the Kaplan–Meier analysis, with patients harboring a pathogenic mutation having a significantly shorter CV event-free survival rate (no somatic myeloid mutation: median event-free survival not reached, with pathogenic mutation: 191.6 months; *p* = 0.026, Figure 2). The median was not reached for patients with or without a CHIP mutation, with the difference in CV event-free survival rate of borderline significance (*p* = 0.079), and for any other somatic myeloid mutation significance was not reached.

**Figure 3:**
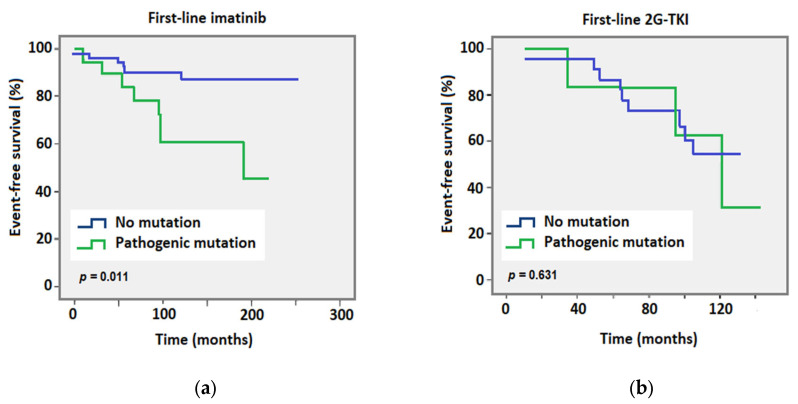
Kaplan–Meier curves of cardiovascular event-free survival for the whole cohort comparing patients who received (**a**) imatinib or (**b**) 2G-TKI in the first line. Data were stratified according to the absence (blue line) or presence (green line) of a pathogenic somatic myeloid mutation. Time was calculated from the date of diagnosis to the date of a cardiovascular event or the date of the last follow-up. Significance was determined using the log-rank test.

The presence of a pathogenic somatic myeloid mutation had a shortening effect on the CV event-free survival rate for patients receiving imatinib in the first line (Figure 3, *p* = 0.011), but not for patients receiving a 2G-TKI (*p* = 0.631). Specifically, for patients who received first-line imatinib, the median event-free survival rate was reduced from 317 months without a mutation to 195 months with a pathogenic somatic myeloid mutation (CV event-free rate of 87.3% vs. 60.4% after 120 months, respectively), while for 2G-TKI, the event-free survival rate was reduced from 133 months without a mutation to 127 months with a mutation (CV event-free rate of 54.1% vs. 31.3% after 120 months, respectively, Table 3).

Considering the CV event-free survival rate according to the TKI received at the time of the event, the presence of a somatic pathogenic myeloid mutation was of borderline significance for patients who received imatinib at the time of the event (*p* = 0.092) but did not reach statistical significance for patients receiving a 2G-TKI at the time of the event (results not shown).

## 4. Discussion

In CML, molecular studies are beginning to reveal genetic profiles associated with resistance to TKI and the risk of progression to blast crisis [20,21,23]. However, only a handful of studies have evaluated the molecular factors associated with the development of CVEs induced by TKIs in CML. In this retrospective study of 102 CML patients, pathogenic myeloid mutations were detected in less than a quarter of the diagnostic samples of CML patients analyzed (24.5%), increasing to 40.2% when variants of uncertain significance were also included, consistent with previous studies [19,20,21,24]. Some variants of possible germline origin were identified but not confirmed in a non-hematopoietic sample, thus, they were excluded from the statistical analysis.

Specifically, the presence of a somatic myeloid mutation was shown to be a significant risk factor for the development of AOE/VTE while on TKI treatment. Coronary syndrome was the most common AOE induced by TKI, followed by ischemic stroke, which constituted over 19% of reported CVEs. The latter is in contrast to a previous study of CVEs in a cohort of 58 CML patients that reported no ischemic strokes [28].

Although we only observed a marginal association of CVE with CHIP mutations, the vast majority of pathogenic variants of uncertain significance were detected in the DTA genes. Our results are in concordance with those of Hadzijusufovic et al. who studied 36 nilotinib-treated CML patients and observed a higher frequency of DTA mutations in those patients who developed AOEs during nilotinib treatment compared to those who did not (65% vs. 32%, *p* < 0.05) [29], although the mutations were detected in samples at the time of best response rather than at diagnosis.

As expected, the CV event-free survival rate was significantly shorter for patients aged ≥ 60 years [30]. Importantly, by age 70 and over, 10–20% of individuals are expected to have CHIP. Advanced age was confirmed as a risk factor by a multivariable analysis, but it is important to highlight that somatic myeloid mutation was an additional independent risk factor, as confirmed by the age-matched case-control study.

Although not the objective of this study, first-line imatinib treatment was a protective factor for CVE when compared to dasatinib and particularly nilotinib (either compared individually or collectively as 2G-TKI), in accordance with the low incidence of CVEs identified in randomized trials [31,32]. It is hard to form conclusions about the effect of dasatinib treatment on CVE risk as only seven patients received it in the first line and the patients were predominantly male with a high incidence of prior CVEs in their medical history. Nevertheless, CVE incidence for first-line dasatinib in this work was higher than the incidence of arterial ischemic events (5%) reported from the DASISION trial [6], and in accordance with observations from a study that analyzed 106 patients who received dasatinib in prospective clinical trials and reported that 14% experienced an arteriothrombotic adverse event [32].

The CVE risk of patients receiving bosutinib or ponatinib could not be evaluated since they were only received by one patient each in the second line (and none in the first line). As such, it remains to be determined whether myeloid mutations can increase the risk of CVE for patients who receive first-line bosutinib, generally associated with low rates of CVE [7,33]. Such studies might be more difficult for patients on ponatinib, who have often received multiple lines of treatment and, thus, would have a confounding effect. Nevertheless, no significant difference in the risk of CVE was observed for patients in this cohort who received more than three lines of TKI treatment.

The detection of a pathogenic somatic myeloid mutation at diagnosis was a significant CVE risk factor for patients receiving first-line imatinib and first-line 2G-TKI but failed to reach significance for patients receiving imatinib or 2G-TKI at the time of the CVE, perhaps due to a multiple TKI line confounding effect of CVE risks. The presence of a myeloid mutation at diagnosis also significantly shortened the CV event-free survival rate for patients receiving first-line imatinib treatment.

In this work, we have considered mutations to the *DNMT3A, TET2, ASXL1,* and *JAK2* genes collectively as CHIP mutations. Mutations in these individual genes were associated with a 1.7-fold, 1.9-fold, 2.0-fold, and 12-fold increased risk of developing coronary heart disease, respectively [16]. The link between CHIP, inflammation, and atherosclerosis is now well-characterized [18,34]. In a previous study, we reported a similar age-independent association between the presence of mutations in DTA genes and an increased risk of vascular events for patients with PV, a *BCR::ABL1*-negative MPN [35]. In addition, and specifically in CML, one study of nilotinib-treated patients found that those who harbored a polymorphism in the lectin-like oxidized LDL receptor gene *LOX1* were at higher risk of developing a CVE (86% with polymorphism had a CVE vs. 35% without *LOX1* polymorphism), presumably due to an induced inflammatory state (atherogenesis) [36]. Thus, our work and other genetic studies have shown that genetic conditions can further influence the CV risk.

Multiple CV risk factors, including classical risk factors such as age and comorbidities (hypertension, diabetes mellitus, etc.), as well as CHIP (or other genetic predisposing factors), may co-exist in an individual CML patient and have a synergistic effect. Although patients with vascular events in their medical history presented a tendency for a higher incidence of myeloid mutations, these differences were not statistically significant. This might be due to the small sample size (only 18/102 patients had a prior CVE in their medical history) or an additional TKI-related effect since certain TKIs are associated with a higher risk of CVEs. For example, first-line nilotinib might not be the most appropriate choice for a patient with a prior AOE [4]. It is worth noting that the median CV event-free survival rate observed for patients who received first-line imatinib, albeit reduced by a myeloid mutation, was still over 16 years. Nevertheless, cardiotoxicity is an important issue given the associated mortality of CVEs, the long-term nature of TKI treatment, and the association of CVEs with the increased patient age (as well as the age-related effect of CHIP). First-line TKI choice is especially important nowadays as many clinicians give upfront 2G-TKIs in order to improve treatment-free remission possibilities. All of these factors should be taken into account when considering first-line TKI choice as well as any future line changes.

To this end, a CV risk-stratification score would be useful to identify patients at a high risk of CVE on TKI. Some groups have demonstrated that the Systematic Coronary Risk Evaluation (SCORE) can predict the CVE risk for patients treated with nilotinib in the first or second line, as well as ponatinib [37]. The HFA/ICOS was also shown to risk stratify patients before the start of TKI [28]. Based on our results, we suggest that the detection of a pathogenic somatic myeloid mutation (particularly a mutation associated with CHIP) can be considered an additional risk factor that could be taken into account when choosing the most appropriate TKI. The incorporation of biomarkers associated with the development of CVEs would help personalize and refine the risk scores to guide clinicians when choosing the most appropriate TKI, although validation in large prospective CML patient cohorts would be required. High-risk patients could benefit from interventions such as increased patient monitoring (e.g., lipid profiles, blood pressure, electrocardiography, and ankle-brachial index measurement), dose modifications, and perhaps aspirin prophylaxis [38].

The limitations of this work include the retrospective nature of the study and that neither lifestyle factors, such as smoking, nor TKI dose were considered, since lower doses may reduce the risk of CVEs [39,40]. However, the key limitation of this study is that the clonal dynamics of mutations were not analyzed during follow-up to monitor the TKI treatment effects. It will be important to carry out NGS in paired samples for a longitudinal analysis of the persistence or clearance of the identified variants [19]. However, the results from other recent molecular studies in CML have also shown that mutations detected at diagnosis were prognostic. For instance, mutations at diagnosis were associated with a risk of relapse upon TKI discontinuation [24], and a higher risk of treatment failure and progression [41], particularly for patients who received imatinib, in accordance with our observations.

It remains to be determined how a variant detected at diagnosis could alter the future risk of CV disease. Based on our results, one hypothesis is that preexisting CHIP confers a basal inflammatory state and susceptibility for atherosclerosis that may be further antagonized by TKI treatment. This could explain the minimal effect of myeloid mutations on CV event-free survival rates for patients who received first-line nilotinib (of less than 2 months), as nilotinib is already proatherogenic, in contrast to imatinib [42].

## 5. Conclusions

We show that the presence of a myeloid mutation at diagnosis is a significant risk factor for CV events on TKI. Importantly, our work shows that the low risk of CVE, traditionally associated with first-line imatinib, was increased by the presence of myeloid mutations as well as older age. Elucidation of the molecular factors predisposing to CVE development in patients with CML is an important area of unmet clinical need due to long TKI exposure.

## Figures and Tables

**Table 1:** Cardiovascular (CV) events registered in CML patients on tyrosine kinase inhibitor (TKI) treatment.

Patient	First CV Event	Second CV Event
1	Deep vein thrombosis	
2	Ischemic stroke	
3	Chronic ischemic syndrome	
4	Acute coronary syndrome	
5	Ischemic stroke	
6	Portal vein thrombosis	Subclavian artery thrombosis
7	Coronary artery disease	
8	Chronic ischemic syndrome	
9	Transient ischemic attack	Transient ischemic attack
10	Peripheral artery disease and acute coronary syndrome	
11	Acute coronary syndrome	
12	Acute coronary syndrome	
13	Ischemic stroke	
14	Portal vein thrombosis	
15	Chronic ischemic syndrome	
16	Ischemic stroke	
17	Acute coronary syndrome	
18	Coronary artery disease	
19	Aortic aneurysm	
20	Acute coronary syndrome	
21	Acute coronary syndrome	
22	Acute coronary syndrome	Acute coronary syndrome
23	Acute coronary syndrome	
24	Acute coronary syndrome	
25	Acute coronary syndrome	
26	Pulmonary embolism ^1^	

^1^ Event related to surgery, not TKI treatment.

**Table 2:** Cox regression multivariable analysis for association with a CV event while on tyrosine kinase inhibitor (TKI) treatment for the cohort of 102 CML patients. Significant values are shown in bold.

	HR	CI	*p*-Value		HR	CI	*p*-Value
Age ≥ 60 years at diagnosis	3.60	1.55–8.33	**0.003**	Age ≥ 60 years at diagnosis	4.11	1.75–9.71	**0.001**
Pathogenic mutation	2.79	1.27–6.10	**0.01**	Any mutation ^1^	2.54	1.15–5.62	**0.021**
First-line imatinib	0.29	0.13–0.64	**0.002**	First-line imatinib	0.31	0.14–0.70	**0.004**
Age ≥ 60 years at diagnosis	3.29	1.42–7.63	**0.005**	Age ≥ 60 years at diagnosis	3.73	1.59–8.77	**0.003**
Pathogenic mutation	2.62	1.20–5.75	**0.016**	Any mutation ^1^	2.49	1.13–5.52	**0.024**
First-line nilotinib	2.38	1.04–5.41	**0.039**	First-line nilotinib	2.16	0.95–4.95	**0.067**
Age ≥ 60 years at diagnosis	3.60	1.55–8.33	**0.003**	Age ≥ 60 years at diagnosis	4.11	1.75–9.71	**0.001**
Pathogenic mutation	2.79	1.27–6.10	**0.010**	Any mutation ^1^	2.53	1.15–5.62	**0.021**
2G-TKI in first line	3.48	1.57–7.75	**0.002**	2G-TKI in first line	3.17	1.43–7.04	**0.004**

^1^ Somatic variants of uncertain significance were also included but potential germline variants were excluded. 2G-TKI: second-generation tyrosine kinase inhibitors (nilotinib and dasatinib); HR: hazard ratio; CI: 95% confidence interval. The multivariable analysis was only carried out for variables with statistical significance (or borderline significance) in the univariable analysis (Supplementary Table S6). The following variables could not be entered into the multivariable analysis together: first-line imatinib with first-line nilotinib or 2G-TKI in the first line, and pathogenic somatic myeloid mutation with any somatic myeloid mutation. Thus, the variables were analyzed in blocks of two sequential multivariable analyses. First-line dasatinib was not evaluated as a variable since it was only received by seven patients.

**Table 3:** Proportion of patients with cardiovascular (CV) event-free survival (%) comparing those with and without a pathogenic somatic myeloid mutation detected at diagnosis who received imatinib or 2G-TKI in the first line.

CV Event-Free Survival (%)	Imatinib		2G-TKI	
	No Mutation	With Mutation	No Mutation	With Mutation
24 months	96.2	94.7	95.8	83.3
48 months	94.1	83.6	91.3	62.5
120 months	87.3	60.4	54.1	31.3

## Data Availability

The data that support the findings of this study are available from the corresponding author upon reasonable request.

## Supplementary Materials

The following supporting information can be downloaded at: https://www.mdpi.com/article/10.3390/cancers15133384/s1, Figure S1: Kaplan–Meier curve of cardiovascular event-free survival for the whole cohort comparing patients who received imatinib (blue line), nilotinib (green line) or dasatinib (yellow line) in the first-line; Table S1: Characteristics of the cohort of 102 CML patients; Table S2: Time (months) of tyrosine kinase inhibitor (TKI) treatment lines received by the CML patients who had a cardiovascular event after TKI start; Table S3: Considering the total number of patients who received imatinib in any line (*n* = 75) or second-generation TKI in any line (2G-TKI, *n* = 53), chi-squared univariable analysis of risk of CV event according to TKI received at the time of CV event; Table S4: Frequency (and %) of variables at diagnosis for patients treated with first-line imatinib, nilotinib or dasatinib; Table S5: List of myeloid mutations detected; Table S6: Chi-squared univariable analysis for association with CV event while on tyrosine kinase inhibitor (TKI) treatment for the cohort of 102 CML patients; Table S7: Chi-squared univariable analyses of associations between presence of myeloid mutations and any cardiovascular (CV) event in medical history for the cohort of 102 CML patients; Table S8: Comparison of variables for CML patients who received first-line imatinib (*n* = 72) with a cardiovascular event on TKI (event) and patients with no event on TKI treatment (no event); Table S9: Comparison of CML patients with a cardiovascular event on TKI (event) and patients with no event on TKI treatment (no event) from the case control study (*n* = 42); Table S10: Cox regression multivariable analyses for associations with cardiovascular event while on tyrosine kinase inhibitor (TKI) treatment for the patients in the case-control study (sex- and age-matched, *n* = 42).
