# Supplementary material for: Presence of Myeloid Mutations in Patients with Chronic Myeloid Leukemia Increases Risk of Cardiovascular Event on Tyrosine Kinase Inhibitor Treatment
Source: Cancers (Basel). 2023 Jun 28;15(13):3384. doi: 10.3390/cancers15133384 (PMC10341219; doi:10.3390/cancers15133384)

Supplementary Table S1. Characteristics of the cohort of 102 CML patients.

Variable	N (%) [range]
Male gender	61 (59.8%)
Age at diagnosis:	
Average	58.1 [13–98]
Median	59.6
≥ 60 years	51 (50.0%)
First-line TKI:	
Imatinib	72 (70.6%)
Dasatinib	7 (6.9%)
Nilotinib	23 (22.5%)
Second-line TKI:	
Imatinib	3 (9.4%)
Dasatinib	14 (43.8%)
Nilotinib	13 (40.6%)
Bosutinib	1 (3.1%)
Ponatinib	1 (3.1%)
Third-line TKI:	
Dasatinib	8 (50.0%)
Nilotinib	3 (18.8%)
Bosutinib	3 (18.8%)
Ponatinib	1 (6.3%)
Asciminib	1 (6.3%)
≥ 3 lines of TKI received	15 (14.7%)
Median TKI exposure (months)	87.5 [36.0–267.0]
Mutation in *BCR::ABL1*	7 (6.9%)
CV event prior to TKI start	18 (17.6%)
CV event on TKI treatment:	26 (25.5%)
Arterial	21 (80.8%)
Venous	5 (19.2%)
Second CV event on TKI treatment	3 (11.1%)
Mutations:	
Pathogenic mutation	25 (24.5%)
CHIP mutation	19 (18.6%)
Any additional mutation[1]	41 (40.2%)
No mutation	77 (75.5%)
Progression to advanced or blast phase	4 (3.9%)

[1] variants of uncertain significance were also included

CV: cardiovascular; CHIP mutation: *DNMT3A, TET2, ASXL1, JAK2* genes; TKI: tyrosine kinase inhibitor

Supplementary Table S2. Time (months) of tyrosine kinase inhibitor (TKI) treatment lines received by the CML patients who had a cardiovascular event after TKI start.

№	Total time on TKI to event (months)	TKI at event	Line at event	First-line TKI	First-line TKI (months)	Second-line TKI	Second-line TKI (months)	Third-line TKI	Third-line TKI (months)
1	122	Imatinib	1						
2	143	Nilotinib	1						
3	57	Imatinib	1						
4	105	Nilotinib	1						
5	192	Nilotinib	2	Imatinib	97	Nilotinib	146		
6	95	Nilotinib	1						
7	68	Nilotinib	1						
8	120	Nilotinib	1						
9	52	Nilotinib	1						
10	100	Nilotinib	1						
11	64	Imatinib	2	Nilotinib	19	Imatinib	45		
12	49	Nilotinib	1						
13	65	Dasatinib	1						
14	34	Dasatinib	1						
15	54	Imatinib	1						
16	55	Imatinib	1						
17	10	Imatinib	1						
18	10	Dasatinib	1						
19	98	Dasatinib	2	Imatinib	26	Dasatinib	75		
20	64	Nilotinib	1						
21	16	Imatinib	1						
22	97	Dasatinib	2	Imatinib	57	Dasatinib	153		
23	67	Imatinib	1						
24	94	Nilotinib	3	Imatinib	16	Dasatinib	14	Nilotinib	64
25	175	Nilotinib	3	Imatinib	120	Dasatinib	53	Nilotinib	2
26*	101	-	-	Imatinib	101				

* Patient was not receiving TKI treatment at time of event, due to discontinuation. The cardiovascular event was related to surgery.

Supplementary Table S3. Considering the total number of patients who received imatinib in any line (n=75) or second-generation TKI in any line (2G-TKI, n=53), chi-squared univariable analysis of risk of CV event according to the TKI received at the time of CV event. Significant values are shown in bold.

		Cardiovascular event			
**TKI at CV event**		**No**	**Yes**	**Total**	***p*-value**
**Imatinib**	**No**	50 (73.5%)	18 (26.5%)	53 (47.6%)	OR 0.33
	**Yes**	67 (89.3%)	8 (10.7%)	75 (52.4%)	*p* = **0.017**
	**Total**	117 (81.8%)	26 (18.2%)	143 (100%)	CI: 0.13–0.82
**2G-TKI**	**No**	66 (88.0%)	9 (12.0%)	75 (52.4%)	OR 2.44
	**Yes**	51 (75.0%)	17 (25.0%)	68 (47.6%)	*p* = **0.048**
	**Total**	117 (81.8%)	26 (18.2%)	143 (100%)	CI: 1.01–5.93

		Pathogenic somatic myeloid mutation			
		**No**	**Yes**	**Total**	***p*-value**
**Imatinib at time of CV event**	**No**	51 (%)	16 (%)	67 (%)	OR 1.91
	**Yes**	5 (%)	3 (%)	8 (%)	*p* = 0.409
	**Total**	56 (%)	19 (%)	75 (100%)	CI: 0.41–8.90
**2G-TKI at time of CV event**	**No**	28 (%)	8 (%)	36 (%)	OR 3.11
	**Yes**	9 (%)	8(%)	17 (%)	*p* = 0.072
	**Total**	37 (%)	16 (%)	53 (100%)	CI: 0.91–10.69

Supplementary Table S4. Frequency (and %) of variables at diagnosis for patients treated with first-line imatinib, nilotinib or dasatinib.

**Variable**	**Imatinib (n=72)**	**Nilotinib (n=23)**	**Dasatinib (n=7)**	***p*-value**
Age	60	53	54	0.166
Gender (female)	30 (41.7)	10 (43.5)	1 (14.3)	0.353
Previous event	14 (19.4)	1 (4.4)	2 (28.6)	0.167
Pathogenic mutation	20 (27.8)	5 (21.7)	1 (14.3)	0.522
CHIP mutation	15 (20.8)	3 (13.0)	1 (14.3)	0.680
Any mutation	29 (40.3)	10 (43.5)	2 (28.6)	0.785

P-values determined using an unpaired ANOVA for mean comparison.

Supplementary Table S5. List of myeloid mutations detected. Pathogenicity was determined according to the Association for Molecular Pathology guidelines [27].

Patient	Gene	cDNA	Protein	VAF	Pathogenicity		Gene	cDNA	Protein	VAF	Pathogenicity
7	*ASXL1*	c.2392_2393del	p.(Ser798*)	46.1	Likely pathogenic						
10	*ASXL1*	c.4170_4171del	p.(His1390Glnfs*33)	27	Likely pathogenic						
22	*ASXL1*	c.2290delC	p.(Leu764Tyrfs*8)	29.7	Pathogenic						
28	*ASXL1*	c.1934delG	p.(Gly645Valfs*58)	4	Likely pathogenic						
38	*ASXL1*	c.1934dupG	p.(Gly646Trpfs*12)	34.4	Likely pathogenic						
64	*ASXL1*	c.1730C>G	p.(Ser577*)	10.2	Likely pathogenic						
71	*ASXL1*	c.1934dupG	p.(Gly646Trpfs*12)	26.9	Pathogenic						
99	*ASXL1*	c.2100_2104del insGA	p.(Tyr700*)	2.8	Likely pathogenic						
100	*ASXL1*	c.2773C>T	p.(Gln925*)	3.6	Pathogenic		*SF3B1*	c.1974G>T	p.(Trp658Cys)	2.1	Likely pathogenic
83	*ASXL1*	c.1815C>A	p.(Cys605*)	2.7	Likely pathogenic		*CEBPA*	c.311_313del	p.(Gly104del)	2.9	VUS
52	*ASXL1*	c.3077del	p.(Gly1026Aspfs*21)	49.7	Likely pathogenic		*JAK2*	c.1177C>G	p.(Leu393Val)	47.1	VUS
68	*ASXL1*	c.1900_1922del	p.(Glu635Argfs*15)	9.2	Pathogenic		*ASXL1*	c.2077C>T	p.(Arg693*)	8	Pathogenic
84	*DNMT3A*	c.1554+1G>A	p.(?)	2.0	Pathogenic		*JAK2*	c.1711G>A	p.(Gly571Ser)	48.8	VUS
79	*DNMT3A*	c.1903C>T	p.(Arg635Trp)	4	Pathogenic						
85	*DNMT3A*	c.2645G>A	p.(Arg882His)	49.9	Pathogenic						
93	*DNMT3A*	c.2206C>T	p.(Arg736Cys)	2.5	Likely pathogenic						
69	*DNMT3A*	c.2377T>G	p.(Tyr793Asp)	21.9	Likely pathogenic						
101	*DNMT3A*	c.1792C>T	p.(Arg598*)	47.4	Pathogenic						
2	*TET2*	c.2148dupA	p.(His717Thrfs*6)	23.3	Likely pathogenic						
25	*TET2*	c.5374delC	p.(His1792Thrfs*28)	8.8	Likely pathogenic						
30	*RUNX1*	c.1023dupC	p.(Ile342Hisfs*?)	4.4	Likely pathogenic						
102	*RUNX1*	c.299C>T	p.(Ser100Phe)	42.1	Likely pathogenic						
17	*KRAS*	c.35G>T	p.(Gly12Val)	23.6	Pathogenic		*WT1*	c.1090_1093dup TCGG	p.(Ala365Valfs* 4)	22.1	Pathogenic
37	*CBL*	c.1210T>C	p.(Cys404Arg)	47.6	Pathogenic		*U2AF1*	c.101C>T	p.(Ser34Phe)	49.8	Pathogenic
77	*IDH2*	c.419G>A	p.(Arg140Gln)	2.6	Pathogenic						

49	*KIT*	c.2429A>T	p.(Asp810Val)	3.6	Likely pathogenic						
33	*ASXL1*	c.3743C>A	p.(Ala1248Asp)	48.7	VUS						
3	*TET2*	c.5939C>T	p.(Thr1980Ile)	50.4	VUS						
14	*TET2*	p.Gln1548del	p.(Gln1548del)	2.6	VUS						
46	*TET 2*	c.1299A>C	p.(Glu433Asp)	49.9	VUS						
59	*TET2*	c.4624_4626del	p.(Gln1542del)	3.2							
60	*TET2*	c.4624_4626del	p.(Gln1542del)	2.5							
72	*TET2*	c.550G>C	p.(Glu184Gln)	25.1	VUS						
16	*JAK2*	c.3323A>G	p.(Asn1108Ser)	51.3	VUS						
27	*JAK2*	c.1177C>G	p.(Leu393Val)	48.1	VUS						
35	*JAK2*	c.1177C>G	p.(Leu393Val)	48.1	VUS						
24	*TP53*	c.406C>T	p.(Thr312Ser)	9.3	VUS						
73	*TP53*	c.642T>G	p.(His214Gln)	30.5	VUS						
15	*JAK2*	c.3323A>G	p.(Asn1108Ser)	51.1	VUS		*CEBPA*	c.207_211del insCTCGCACC	p.(Pro70_Ser71 delinsSerHisPro)	2.6	VUS
12	*JAK2*	c.1177C>G	p.(Leu393Val)	49.8	VUS		*EZH2*	c.1209_1211del	p.(Glu404del)	2.0	VUS
82	*EZH2*	c.1209_1211del	p.(Glu404del)	3.2	VUS						
47	*NPM1*	c.847-3dupC	p.(?)	5.5	VUS						
55	*CALR*	c.1191_1199del	p.(Glu398_Asp400del)	52.9	VUS						

VAF: variant allele frequency; VUS: variant of uncertain significance

Transcript IDs (reference genome GRCh37/hg19): ASXL1 NM_015338, CALR NM_004343, CBL NM_005188, CEBPA NM_004364, DNMT3A NM_022552, EZH2 NM_004456, IDH2 NM_002168, JAK2 NM_004972, KIT NM_000222, KRAS NM_004985, NPM1 NM_002520, RUNX1 NM_001754, SF3B1 NM_012433, TET2 NM_001127208, TP53 NM_000546, U2AF1 NM_006758, WT1 NM_024426.

Supplementary Table S6. Chi-squared univariable analysis for association with CV event while on tyrosine kinase inhibitor (TKI) treatment for the cohort of 102 CML patients. Significant values are shown in bold.

		**Cardiovascular event on TKI treatment**			
		**No**	**Yes**	**Total**	***p*-value**
**Age ≥ 60 years at diagnosis**	**No**	43 (84.3%)	8 (15.7%)	51 (50.0%)	**OR: 2.93**
	**Yes**	33 (64.7%)	18 (35.3%)	51 (50.0%)	**p = 0.04**
	**Total**	76 (74.5%)	26 (25.5%)	102 (100%)	**CI: 1.14–7.57**
**Gender (male)**	**No**	32 (78.0%)	9 (22.0%)	41 (40.2%)	OR: 0.73
	**Yes**	44 (72.1%)	17 (27.9%)	61 (59.8%)	p = 0.644
	**Total**	76 (74.5%)	26 (25.5%)	102 (100%)	CI: 0.29–1.84
**Previous CV event**	**No**	63 (74.1%)	22 (25.9%)	85 (82.4%)	OR: 0.88
	**Yes**	13 (76.5%)	4 (23.5%)	17 (16.7%)	p = 1.000
	**Total**	76 (74.5%)	26 (25.5%)	102 (100%)	CI: 0.26–2.99
**Pathogenic mutation**	**No**	62 (80.5%)	15 (19.5%)	77 (75.5%)	**OR 3.25**
	**Yes**	14 (56.0%)	11 (44.0%)	25 (24.5%)	**p = 0.019**
	**Total**	76 (74.5%)	26 (25.5%)	102 (100%)	**CI: 1.23–8.57**
**CHIP mutation**	**No**	65 (78.3%)	18 (21.7%)	83 (81.4%)	OR 2.63
	**Yes**	11 (57.9%)	8 (42.1%)	19 (18.6%)	p = 0.082
	**Total**	76 (74.5%)	26 (25.5%)	102 (100%)	CI: 0.92–7.50
**Any mutation**[1]	**No**	50 (82.0%)	11 (18.0%)	61 (59.8%)	**OR 2.62**
	**Yes**	26 (63.4%)	15 (36.6%)	41 (40.2%)	**p = 0.040**
	**Total**	76 (74.5%)	26 (25.5%)	102 (100%)	**CI: 1.06– 6.52**
***BCR::ABL1* mutation**	**No**	8 (80%)	2 (20%)	10 (76.9%)	OR: 0.73
	**Yes**	3 (100%)	0 (0%)	3 (23.1%)	p = 1.000
	**Total**	11 (84.6%)	2 (15.4%)	13 (100%)	CI: 0.51–1.04
**≥ 3 TKI lines**	**No**	66 (75.9%)	21 (24.1%)	87 (85.3%)	OR: 1.57
	**Yes**	10 (66.7%)	5 (33.3%)	15 (14.7%)	p = 0.523
	**Total**	76 (74.5%)	26 (25.5%)	102 (100%)	CI: 0.48–5.12
**First-line imatinib**	**No**	18 (60%)	12 (40%)	30 (29.4%)	**OR: 0.36**
	**Yes**	59 (81.9%)	13 (18.1%)	72 (70.6%)	**p = 0.045**
	**Total**	76 (74.5%)	26 (25.5%)	102 (100%)	**CI: 0.14–0.92**
**First-line nilotinib**	**No**	62 (78.5%)	17 (21.5%)	79 (77.5%)	OR: 2.35
	**Yes**	14 (60.9%)	9 (39.1%)	23 (22.5%)	p = 0.093
	**Total**	76 (74.5%)	26 (25.5%)	102 (100%)	CI: 0.87–6.34
**First-line dasatinib**	**No**	72 (75.8%)	23 (24.2%)	95 (93.1%)	OR: 2.35
	**Yes**	4 (57.1%)	3 (42.9%)	7 (6.9%)	p = 0.286
	**Total**	76 (74.5%)	26 (25.5%)	102 (100%)	CI: 0.49–11.27

P-values determined using Fisher Exact test.

OR: odds ratio, CI: 95% confidence interval, CHIP mutation: *DNMT3A, TET2, ASXL1, JAK2* genes; TKI: tyrosine kinase inhibitor.

Supplementary Table S7. Chi-squared univariable analyses of associations between presence of myeloid mutations and any cardiovascular (CV) event in medical history for the cohort of 102 CML patients. Significant values are shown in bold.

**Cardiovascular event in medical history (including before TKI start)**					
		**No**	**Yes**	**Total**	***p*-value**
**Pathogenic mutation**	**No**	50 (64.9%)	27 (35.1%)	77 (75.5%)	OR 1.71
	**Yes**	13 (52.0%)	12 (48.0%)	25 (24.5%)	*p* = 0.344
	**Total**	63 (61.8%)	39 (38.2%)	102 (100%)	CI: 0.69–4.26
**CHIP mutation**	**No**	53 (63.9%)	30 (36.1%)	83 (81.4%)	OR 1.59
	**Yes**	10 (52.6%)	9 (47.4%)	19 (18.6%)	*p* = 0.435
	**Total**	63 (61.8%)	39 (38.2%)	102 (100%)	CI: 0.58–4.35
**Any mutation**[1]	**No**	40 (65.6%)	21 (34.4%)	61 (56.8%)	OR 1.49
	**Yes**	23 (56.1%)	18 (43.9%)	41 (43.2%)	*p* = 0.407
	**Total**	63 (61.8%)	39 (38.2%)	102 (100%)	CI: 0.66–3.66

[1] variants of uncertain significance were also included

OR: odds ratio; CI: 95% confidence interval; CHIP mutation: *DNMT3A, TET2, ASXL1, JAK2* genes; TKI: tyrosine kinase inhibitor.

Supplementary Table S8. Comparison of variables for CML patients who received first-line imatinib (n=72) with a cardiovascular event on TKI (event) and patients with no event on TKI treatment (no event).

		**Event**	**No event**	***p*-value**
**Age at diagnosis**	N	13	59	0.053
	Mean	68.8	58.3	
	SE	2.42	2.56	
**Pathogenic mutation**	N	13	59	**0.001**
	Mean	0.62	0.19	
	SE	0.05	0.14	
**Additional mutation**[1]	N	13	59	**0.003**
	Mean	0.77	0.32	
	SE	0.12	0.06	
**Follow-up time (months)**	N	21	21	0.385
	Mean	160.9	144.1	
	SE	22.6	7.6	

[1] variants of uncertain significance were also included

Means compared using the Student paired T-test of equal variance for unpaired samples. Normality was determined using the Shapiro-Wilk test. Significant values are shown in bold. N: number of data considered, SE: standard error.

Supplementary Table S9. Comparison of CML patients with a cardiovascular event on TKI (event) and patients with no event on TKI treatment (no event) from the case control study (n=42).

		Event	No event	*p*-value
**Age at diagnosis**	N	21	21	
	Mean	62.8	63.0	
	Median	63.5	65.0	
	Range	40.0–84.0	41.0–83.7	
	Normality	0.165	<0.001	0.967
**Pathogenic mutation**	N	10	3	
	Mean	0.48	0.14	**0.019**
**Additional mutation**[1]	N	13	5	
	Mean	0.62	0.24	**0.012**
**Follow-up time (months)**	N	21	21	
	Mean	146.9	152.8	
	Median	131.2	153.7	
	Range	36.4–317.0	91.0–250.9	
	Normality	<0.001	<0.001	0.748
**First-line imatinib**	N	11	17	
	Mean	0.57	0.76	0.199
**First-line nilotinib**	N	8	3	
	Mean	0.62	0.29	**0.030**
**First-line dasatinib**	N	2	1	
	Mean	0.38	0.19	0.180
**≥ 3 TKI lines**	N	5	2	
	Mean	0.24	0.10	0.224

[1] variants of uncertain significance were also included

Means compared using the Student paired T-test of equal variance for unpaired samples. Normality was determined using the Shapiro-Wilk test. Significant values are shown in bold. N: number of data considered, TKI: tyrosine kinase inhibitor

Supplementary Table S10. Cox regression multivariable analyses for associations with cardiovascular event while on tyrosine kinase inhibitor (TKI) treatment for the patients in the case-control study (sex- and age-matched, n=42).

	OR	CI	*p*-value
**BLOCK 1**			
**Any mutation[1]**	2.61	1.01–6.73	**0.047**
**First-line imatinib**	0.19	0.06–0.56	**0.003**
**BLOCK 2**			
**Any mutation[1]**	2.72	1.06–6.99	**0.037**
**First-line nilotinib**	3.17	1.11–9.01	**0.03**
**BLOCK 3**			
**Any mutation[1]**	2.35	1.08–5.13	**0.033**
**2G-TKI in first-line**	2.61	1.01–6.73	**0.049**

Significant values are shown in bold. The following variables could not be entered into the multivariable analysis together: First-line imatinib with first-line nilotinib, and first-line nilotinib with 2G-TKI in first-line; thus the variables were analyzed in blocks of sequential multivariable analyses. First-line dasatinib was not included in the multivariable analysis as it was received by too few patients (n=2 in case-control).

[1] variants of uncertain significance were also included.

TKI: tyrosine kinase inhibitor; 2G-TKI: second-generation TKI (nilotinib or dasatinib); OR: odds ratio; CI: 95% confidence interval.

Supplementary Figure S1. Kaplan–Meier curve of cardiovascular event-free survival for the whole cohort comparing patients who received imatinib (blue line), nilotinib (green line) or dasatinib (yellow line) in the first-line. Time calculated from date of diagnosis to date of cardiovascular event or date of last follow-up. Significance determined using the Log Rank test.

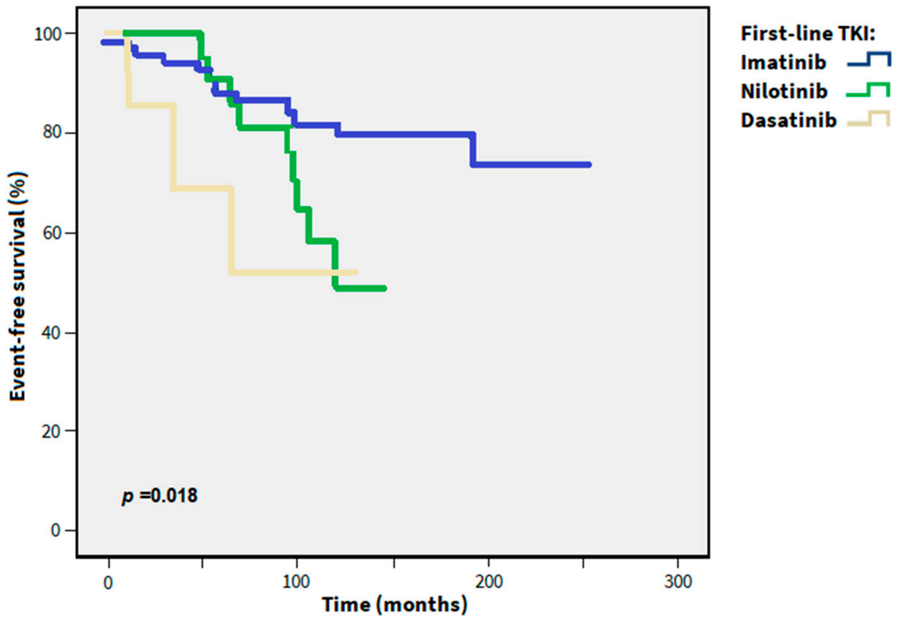

Supplement: Supplementary file 1 [file cancers-15-03384-s001.zip › cancers-2437181-supplementary.pdf]
